# Small intestine histomorphometry of beef cattle with divergent feed efficiency

**DOI:** 10.1186/1751-0147-55-9

**Published:** 2013-02-05

**Authors:** Yuri Montanholi, Ananda Fontoura, Kendall Swanson, Brenda Coomber, Shigeto Yamashiro, Stephen Miller

**Affiliations:** 1 Department of Animal and Poultry Science, University of Guelph, 70-50 Stone Road East, Guelph, N1G 2W1, ON, Canada; 2 Universidade Federal do Pará, Avenida Universitária s/n, 68745-000, Castanhal, Pará, Brasil; 3 Department of Animal Sciences, North Dakota State University, PO box 6050, Fargo, 58108-6050, ND, USA; 4 Department of Biomedical Sciences, University of Guelph, 3-50 Stone Road East, Guelph, N1G 2W1, ON, Canada; 5 Livestock Gentec, 1400 College Plaza 8215-112 Street, Edmonton, T6G 2C8, AB, Canada

**Keywords:** Bovine, Duodenum, Functional workload, Ileum, Intestinal epithelium, Intestinal crypts, Residual feed intake

## Abstract

**Background:**

The provision of feed is a major cost in beef production. Therefore, the improvement of feed efficiency is warranted. The direct assessment of feed efficiency has limitations and alternatives are needed. Small intestine micro-architecture is associated with function and may be related to feed efficiency. The objective was to verify the potential histomorphological differences in the small intestine of animals with divergent feed efficiency.

**Methods:**

From a population of 45 feedlot steers, 12 were selected with low-RFI (superior feed efficiency) and 12 with high-RFI (inferior feed efficiency) at the end of the finishing period. The animals were processed at 13.79 ± 1.21 months of age. Within 1.5 h of slaughter the gastrointestinal tract was collected and segments from duodenum and ileum were harvested. Tissue fragments were processed, sectioned and stained with hematoxylin and eosin. Photomicroscopy images were taken under 1000x magnification. For each animal 100 intestinal crypts were imaged, in a cross section view, from each of the two intestinal segments. Images were analyzed using the software ImageJ®. The measurements taken were: crypt area, crypt perimeter, crypt lumen area, nuclei number and the cell size was indirectly calculated. Data were analyzed using general linear model and correlation procedures of SAS®.

**Results:**

Efficient beef steers (low-RFI) have a greater cellularity (indicated by nuclei number) in the small intestinal crypts, both in duodenum and ileum, than less efficient beef steers (high-RFI) (P < 0.05). The mean values for the nuclei number of the low-RFI and high-RFI groups were 33.16 and 30.30 in the duodenum and 37.21 and 33.65 in the ileum, respectively. The average size of the cells did not differ between feed efficiency groups in both segments (P ≥ 0.10). A trend was observed (P ≤ 0.10) for greater crypt area and crypt perimeter in the ileum for cattle with improved feed efficiency.

**Conclusion:**

Improved feed efficiency is associated with greater cellularity and no differences on average cell size in the crypts of the small intestine in the bovine. These observations are likely to lead to an increase in the energy demand by the small intestine regardless of the more desirable feed efficiency.

## Background

One of the major costs in beef production is the provision of feed. Optimizing the production of beef related to the amount fed to animals would bring significant economic [1,2] and environmental benefits [3,4]. The direct assessment of feed efficiency in cattle is one of the ways to reduce those costs of production. However, there are prohibitive limitations (labour, time spent, costs, etc.) for employing this approach in a large scale by the beef industry [5]. Therefore, the identification of indirect predictors of feed efficiency would more easily and economically allow for the assessment of feed efficiency to be readily adopted by the beef industry. As a result, genetic selection and nutritional manipulation for improved feed efficiency could be greatly enhanced. In addition, further studies on the biology associated with feed efficiency would lead to advances in our knowledge about the efficiency of feed utilization by the bovine. Although the specific biological mechanisms that affect feed efficiency have yet to be fully elucidated, it is likely to be controlled by a combination of factors including physiological [6-8], genetic [9-11] and behavioral mechanisms [3,8,12]. Residual feed intake (RFI) is a feed efficiency measure, first defined in beef cattle by [13] and largely used to study the biology of feed efficiency and to verify the effectiveness of indirect indicators of feed efficiency [14-18]. Differences in RFI reflect variation among animals’ background energy requirements, which are largely influenced by the visceral organs [3,19].

The gastrointestinal tract is an important energy sink using a disproportionate amount of energy in proportion to its weight [20]. For instance, when compared with muscle tissue, which accounts for six times more body weight than the gastrointestinal tract, the gastrointestinal tract presents two and half times higher fasting heat production [21]. The gastrointestinal tract appears to alter its mass and metabolism in accordance to dietary intake within and across physiological stages of maintenance, growth, fattening or lactation [22]. The small intestine, in particular, possesses the adaptive capacity to alter form and function in response to changes in digestive demand [23] to reach the nutrient needs for the animal using variable amounts of energy and protein according to the background requirements and production level [24].

It has been described that incremental starvation produces progressive small intestine atrophy in mice [25] and structural changes to the mucosa of rats, which include disappearance of some villi and a reduction in the size and number of crypts [26]. In contrast, studies related to re-feeding and feeding for *ad libitum* intake indicate histological changes in small intestine epithelium [27], while studying the feeding response in starved snakes, reported that the thickness of the intestinal mucosa increased three times after 48–72 h of re-feeding.

Therefore, the small intestine responds rapidly and dramatically to changes in functional workload, such as starvation or feeding for *ad libitum* intake. These changes include modifications in the intestinal micro-architecture. Beef cattle with different feed efficiency substantially differ in the amount of feed consumed to achieve the same productive performance [8,28]. Thus, one can hypothesize that cattle with superior and inferior feed efficiency may have differences in their small intestine architecture, which could be associated with differences in feed intake. The objective of this study was to conduct histomorphometrical evaluation of the bovine small intestine (duodenum and ileum) to characterize the histological patterns in response to divergent feed efficiency.

## Methods

### Animals, experimental design and sample collection

Housing and experimental conditions were previously described in detail by [29]. Briefly, individual feed intake was measured daily during the 140 d of the experiment. Animals were divided in 3 pens of 15 steers each. Animals were weighed and ultrasound was performed, for assessing subcutaneous fat deposition, every 28 d until slaughter. Steers were fed a high-moisture corn-based diet for *ad libitum* intake. Steers were handled and monitored meeting or exceeding the recommendations of the Canadian Council of Animal Care guidelines (1993). All procedure protocols were approved by the University of Guelph’s Animal Care Committee. The determination of RFI was done through a regression of dry matter intake on mid-experiment body weight, average daily gain and end-experiment backfat thickness, as described by [30]. From the population of 45 crossbred steers, the 24 animals with extreme feed efficiency were selected: 12 with high-RFI (inferior feed efficiency) and 12 with low-RFI (superior feed efficiency). Animals were processed at 13.79 ± 1.21 months of age. The gastrointestinal tract was collected within 1.5 h after slaughter; two segments of 20 cm were gently harvested from duodenum (immediately distal to the pylorus) and ileum (immediately proximal to the ileocecal valve) [31].

### Sample processing and histomorphometry

Fragments of duodenum and ileum were first washed in a 0.9% saline solution. Tissue fragments were pinned in cardboard and then fixed in 10% neutral phosphate buffered formalin under moderate agitation for 24 h and processed for 8:45 h in a tissue processor (Renaissance TP™: Ventana Medical Systems Inc.; Tucson, U.S.A.). Fixed samples then were embedded in paraffin. Paraffin blocks were sectioned at 5 μm thickness using a microtome (Shandon Finesse Microtome 325®: Thermo Electron Corporation; Waltham, U.S.A.) and stained with hematoxylin and eosin according to the method described previously by [32].

**Figure 1:**
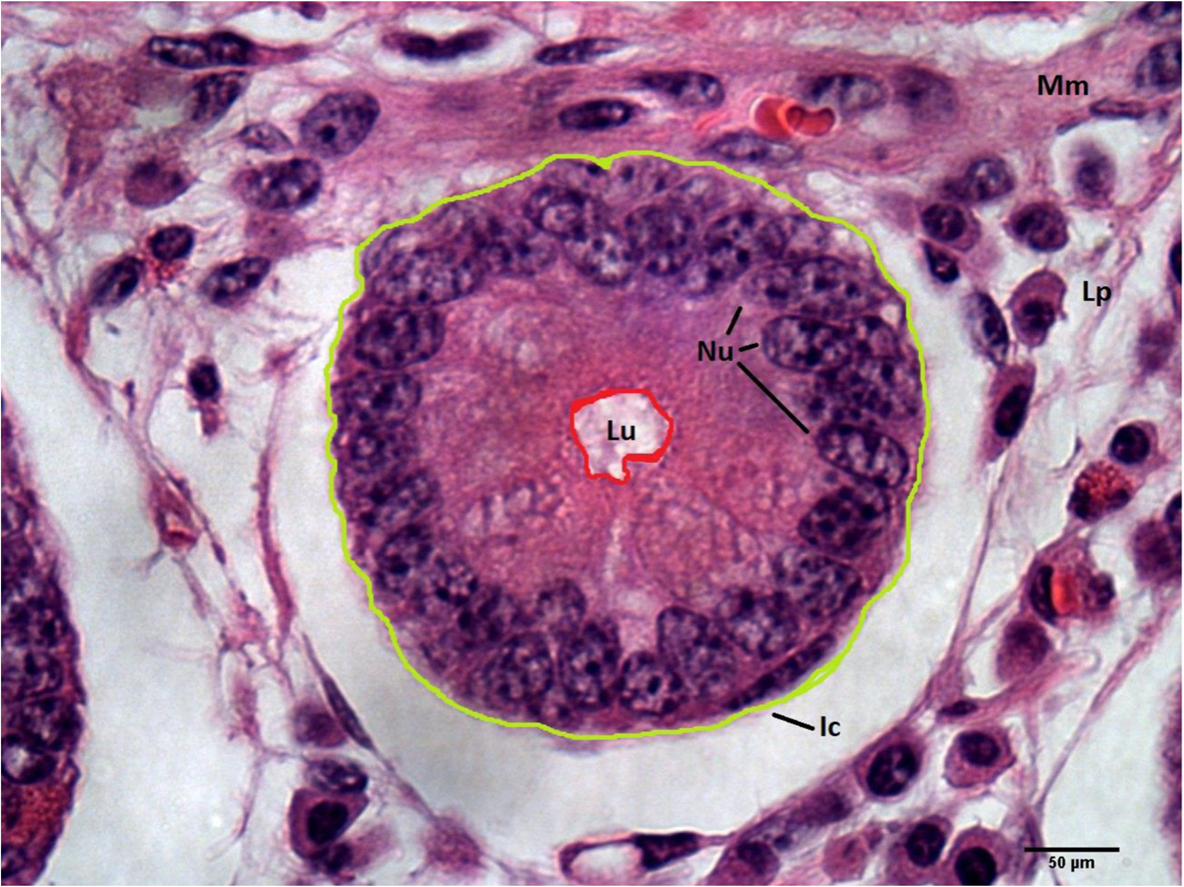
**Light microscopy of an oblique cross section of the intestinal mucosa (1000× capture magnification).** Note the intestinal crypt (Ic) adjacent to the *muscularis mucosae* (Mm), the lumen of the intestinal crypt (Lu), the nuclei of intestinal cells (Nu) around the intestinal crypt and the lamina propria (Lp). The green and red contours were used to obtain measures of the intestinal crypt and the intestinal crypt lumen, respectively.

Histological images were taken using bright field at 1000x magnification (under immersion oil) with a Leica DMLB microscope (Leica Microsystems Inc.®, Wetzlar, Germany) equipped with a video camera QICAM Fast 1394 (Qcapture®, Surrey, BC, Canada) connected to the computer-based image analysis software QImaging (Qcapture®, Surrey, BC, Canada). Histological measurements were made with ImageJ® imaging analysis software (U.S. National Institutes of Health, Bethesda, Maryland, USA). For each steer 100 crypts were measured, in a cross section of both segments (duodenum and ileum), Figure 1. The measurements taken were crypt area (CA; μm^2^), crypt perimeter (CP; μm), crypt lumen area (LA; μm^2^) and nuclei number (NN). In addition, the average cell size (CS; μm^2^) was determined by subtracting the crypt lumen area from the total crypt area and then dividing this value by the nuclei number, which represents the number of mucosal cells present on each transversal image of the crypt (Figure 1). It was observed a separation of the epithelium from the underlying lamina propria, Figure 1. This artifact did not compromised the architecture of the intestinal crypts, which were the target structures for this study. All the pictures were taken and assessments were made by the same observer, who was blinded as to which feed efficiency group the samples belonged to.

### Statistical analysis

Data were analyzed using SAS® software (SAS Institute, Cary, NC, USA). Means of the two feed efficiency groups were tested using the general linear model procedure and compared using T-test, according to the following model:

$$Y_{\text{ij}}=\mu +\text{Grou}p_{i}+\varepsilon_{\text{ij}}$$

where, *Y*_*ij*_ is the dependent variable (RFI and histomorphometrical measures), *μ* is the overall mean effect*; Group*_*i*_ is the fixed effect of feed efficiency group and; *ε*_*ij*_ is the residual error. Pearson correlation was determined within each group using the correlation procedure. For all analyses data were considered statistically significant when P ≤ 0.05 and were considered a trend towards significance when 0.10 ≥ P > 0.05.

## Results

The mean value for the low-RFI and high-RFI groups were −0.53, and 0.64 kg/d (P < 0.001), respectively. This represent a difference in daily dry matter feed intake of 1.17 kg more feed intake for the cattle with inferior feed efficiency to achieve the same performance as the steers with superior feed efficiency without differences on subcutaneous fat deposition, as a result of the adjustment for backfat thickness in the RFI prediction model.

The descriptive statistics composed by mean, standard deviation, coefficient of variation, minimum and maximum values, of the histomorphometrical measures is presented in Table 1. It is interesting to note that the different measurements obtained in both duodenum and ileum presented similar variability, as indicated by the coefficient of variation.

**Table 1:** Descriptive statistics of all traits analyzed

**Segments**	**Trait**	**Mean**	**Standard deviation**	**Coefficient variation (%)**	**Minimum**	**Maximum**
duodenum	crypt area (CA; μm^2^)	3024.0	344.30	11.39	2302.0	3648.0
	crypt perimeter (CP; μm)	199.24	11.75	5.90	173.48	219.48
	cell size (CS; μm^2^)	94.67	7.29	7.70	82.52	107.33
	nuclei number (NN)	31.73	3.60	11.34	25.84	39.73
ileum	crypt area (CA; μm^2^)	2918.0	288.25	9.88	2367.0	3529.0
	crypt perimeter (CP; μm)	199.70	13.35	6.68	177.35	240.38
	cell size (CS; μm^2^)	83.60	6.39	7.64	74.02	97.95
	nuclei number (NN)	35.42	3.52	9.94	29.62	41.83

Table 2 reports the comparisons of the means for all the histomorphometry traits relative to feed efficiency group (low-RFI and high-RFI). Perimeter and area of the crypt (CP; CA) in the duodenum showed a tendency (P ≤ 0.10) to be larger for the more efficient animals (low-RFI group), which was not seen in the ileum crypts (P ≥ 0.10). The cell size (CS) did not differ between the RFI groups in both segments (P ≥ 0.10). Nuclei number (NN) was significantly greater in the low-RFI group than the less efficient animals (high-RFI) in both segments (P ≤ 0.05).

**Table 2:** Mean values by RFI-groups (residual feed intake -high or -low) for intestinal traits

**Segments**	**Trait**	**High-RFI**	**Low-RFI**	**P-value**
duodenum	crypt area (CA; μm^2^)	2916.50	3130.93	0.12
	crypt perimeter (CP; μm)	195.57	202.91	0.12
	cell size (CS; μm^2^)	94.65	94.69	0.98
	nuclei number(NN)	30.30	33.16	0.04
ileum	crypt area (CA; μm^2^)	2857.16	2978.47	0.31
	crypt perimeter (CP; μm)	195.73	203.68	0.14
	cell size (CS; μm^2^)	83.54	83.67	0.96
	nuclei number (NN)	33.65	37.21	0.001

Correlations between the measurements in each segment and within each the RFI groups are shown in Table 3. Negative correlations were observed between CA, CP and NN in duodenum with low-RFI (P ≤ 0.05), the same measures in the ileum were not associated with any of the feed efficiency groups (P ≥ 0.10). Feed efficiency in the less efficient cattle (high-RFI group) appeared to be positively correlated with CS in both duodenum (P ≤ 0.10) and ileum (P ≤ 0.05).

**Table 3:** Correlations of histomorphometry and efficiency by RFI-groups (residual feed intake -high or -low)

**Segments**	**Measurement**	**High-RFI**		**Low-RFI**	
		**r**	**P-value**	**r**	**P-value**
duodenum	crypt area (CA)	0.15	0.61	−0.59	0.04
	crypt perimeter (CP)	0.15	0.63	−0.57	0.04
	cell size (CS)	0.53	0.07	0.08	0.78
	nuclei number (NN)	−0.03	0.91	−0.68	0.01
Ileum	crypt area (CA)	0.34	0.27	−0.16	0.60
	crypt perimeter (CP)	0.29	0.35	−0.21	0.50
	cell size (CS)	0.61	0.01	0.06	0.84
	nuclei number (NN)	−0.05	0.85	−0.17	0.57

## Discussion

The small intestine is an organ with intense metabolic rate, using 17 to 25% of whole-body oxygen consumption [33], with a tremendous capacity to adjust function, size and shape according to the physiological demand in ruminants [22]. The intense metabolic rate of the small intestine is mostly due to the energy expenditure for biochemical processes by the intestinal cells [34] and is also due to constant and continuous epithelium renewal [35-37] to maintain or to cope with variations in workload [38,39]. The later factor is associated with changes in tissue structure [40,41]. Additionally, the workload of the small intestine is particularly increased in cattle fed with diets rich in starch [42] as in the present study.

The similar coefficients of variation, observed on duodenum and ileum measurements, of CA, CP, NN and the CS in the cross section view of the crypt suggests a comparable homogeneity of the same measures in both intestinal segments, which was also observed by other authors [43,44]. This similarity also indicates the consistency of the assessments conducted by a single observer. It is also interesting to notice that the mean values for CS were of larger magnitude in the duodenum in comparison to the ileum. Conversely, the values for NN were higher in the ileum. These results are in agreement with the findings made by [45] studying the cellular dynamics of avian intestine. This author reported that the small intestine possess a negative association between cell size and number of cells in its different segments, where the proximal part (in the case of this study duodenum) had a larger but fewer cells, in contrast to the distal parts, where the ileum could be included, which had smaller but more numerous cells.

The fact that the number of cells, represented by nuclei number, was higher (both in the duodenum and ileum) and the crypt area and perimeter of the duodenum were positively associated with improved feed efficiency, based on the correlation analysis, indicates a more metabolically active small intestine in cattle with superior feed efficiency. Similarly, [24] studying bulls of different breeds, described that a more efficient and higher growth rate breed of cattle had more cells in all small intestinal segments analyzed than the less efficient and lower growth rate cattle breed. We can infer that a greater cellularity and the lack of difference on cell size may associated with larger villi or a more intense reposition of intestinal cells in the villi or both [35], which cannot be distinguished with the present data. Regardless of the nature of such associations, it is strongly indicative that a more metabolic active instestine not only leads to a better absorption of nutrients [46] but also to a better energetic efficiency. In addition, the correlations of histomorphometrical measures in duodenum (CA; CP; NN) and feed efficiency in more efficient beef steers (low-RFI) also support this argument.

Despite the fact that our results for CS did not differ between feed efficiency groups (P ≥ 0.10), a study by [47] described that the small intestine responds to differences in feed intake by altering organ visceral mass via an increase in the size of cells (hyperthophy). We observed a positive correlation between feed efficiency in the high-RFI group and the CS in the ileum, which suggests that an inefficient steer may have larger mucosal cells in this segment. This finding requires further investigations. On the other hand, [48] described that increased intestinal workload through changes on dietary protein level resulted in a quadratic change in the small intestinal mucosa. The DNA concentration increased when the protein levels were 8.5% to 10.7% (dry matter), resulting in a hyperplasia that is in line with the present findings.

The histomorphometric results of this study indicate that more efficient beef steers (low-RFI group) have increased metabolic activity in the small instestine, which is associated with improved feed efficiency in cattle [24] and also in other species [47,49,50]. Despite the fact that this increase is associated with a greater energetic demand [38], increasing the maintenance requirements [51]; the cost-benefit of this more functional small intestine results in more animal growth (productivity) per unit of feed intake. Expenditures with tissue plasticity [52] and cellular biochemical processes in small intestine are known to be largely influenced by the animal’s different physiological states [39] and also by changes according to the level of intake and diet composition via changes in the visceral organ mass [53]. The present study indicates that there is also variation in these expenditures due to individual variation and that such variation is associated with feed efficiency. Finally, the histomorphometrical associations found in here have a potential for further technical improvements (i.e. automated imaging analysis) that may result in a tool for indirectly assessing may feed efficiency in the bovine. This could have immediate applications on breeding programs, where there is a possibility of evaluating the progeny of bulls through sampling their offspring at slaughter.

## Conclusion

There are differences in small intestine micro-architecture of beef cattle with divergent feed efficiency. Improved feed efficiency was associated with greater cellularity in the small intestine crypts and no differences in average cell size, both in duodenum and ileum, as indicated by the nuclei number in the intestinal crypts and the direct associations between crypt area and crypt perimeter with feed efficiency. It is logical to suggest that the benefits of a more metabolically active small intestine are greater than the energetic costs associated with the increased workload, which leads to improved feed efficiency. Further studies aiming to develop imaging analysis techniques for optimizing these measures are warranted and may lead to solutions for improvement of feed efficiency in beef cattle.

## Abbreviations

RFI: Residual feed intake; CA: Crypt area; CP: Crypt perimeter; LA: Crypt lumen area; NN: Nuclei number; CS: Cell size; Mu: Mucosa; Mm: *Muscularis mucosae*; lc: Intestine crypt; Lu: Intestine crypt lumen; Nu: Nuclei.

## Competing interests

The authors declare that they have no competing interests.

## Authors’ contributions

The hypothesis was developed by YM and KS. The study was designed by YM, SM and KS. A pilot study and the development of methodology was done by YM, SY and BC. SY trained YM for tissue processing and slides preparation. YM processed the samples and prepared slides. BC trained AF for microscopy imaging. AF performed the imaging work and measurements and drafted the manuscript. YM performed all statistical calculations. All authors read, revised, provided suggestions and approved the final manuscript.

## References

[B1] Herd RM Archer JA Arthur PF Reducing the cost of beef production through genetic improvement in residual feed intake: opportunity and challenges to application J Anim Sci 2003 81 Suppl. 1 E9 E17

[B2] Maddock TD Lamb GC The Economic Impact of Feed Efficiency in Beef Cattle[http://edis.ifas.ufl.edu/an217]

[B3] Nkrumah JD Okine EK Mathison GW Schmid K Li C Basarab JA Price MA Wang Z Moore SS Relationships of feedlot feed efficiency, performance, and feeding behavior with metabolic rate, methane production, and energy partitioning in beef cattle J Anim Sci 2006 84 145 153 16361501 10.2527/2006.841145x

[B4] Hegarty SR Goopy JP Herd RM McCorkell B Cattle selected for lower residual feed intake have reduced daily methane production J Anim Sci 2007 85 1479 1486 10.2527/jas.2006-236 17296777

[B5] Arthur PF Archer JA Herd RM Feed intake and efficiency in beef cattle: overview of recent Australian research and challenges for the future Aust J Exp Agr 2004 44 361 369 10.1071/EA02162

[B6] Herd RM Oddy VH Richardson EC Biological basis for variation in residual feed intake in beef cattle. 1. Review of potential mechanisms Aust J Exp Agr 2004 44 423 430 10.1071/EA02220

[B7] Richardson EC Herd RM Archer JA Arthur PF Metabolic differences in angus steers divergently selected for residual feed intake Aust J Exp Agr 2004 44 441 452 10.1071/EA02219

[B8] Montanholi YR Swanson KC Palme R Schenkel FS McBride BW Lu D Miller SP Assessing feed efficiency in beef steers through feeding behavior, infrared thermography and glucocorticoids Animal 2010 4 692 701 10.1017/S1751731109991522 22444121

[B9] Schenkel FS Miller SP Wilton JW Genetic parameters and breed differences for feed efficiency, growth, and body composition traits of young beef bulls Can J Anim Sci 2004 84 177 185 10.4141/A03-085

[B10] Chen Y Gondro C Quinn K Herd RM Parnell PF Vanselow B Global gene expression profiling reveals genes expressed differentially in cattle with high and low residual feed intake Anim Genet 2010 42 475 490 21906099 10.1111/j.1365-2052.2011.02182.x

[B11] Mujibi FDN Nkrumah JD Durunna ON Grant JR Mah J Wang Z Basarab J Plastow G Crews DH Jr Moore SS Associations of marker panel scores with feed intake and efficiency traits in beef cattle using preselected single nucleotide polymorphisms J Anim Sci 2011 89 3362 3371 10.2527/jas.2010-3362 21642494

[B12] Bingham GM Friend TH Lancaster PA Carstens GE Relationship between feeding behavior and residual feed intake in growing Brangus heifers J Anim Sci 2009 87 2685 2689 10.2527/jas.2009-1851 19395511

[B13] Koch RM Swiger LA Chambers D Gregory KE Efficiency of feed use in beef cattle J Anim Sci 1963 22 486 494

[B14] Archer JA Arthur PF Herd RM Parnell PF Pitchford WS Optimum postweaning test for measurement of growth rate, feed intake, and feed efficiency in British breed cattle J Anim Sci 1997 75 2024 2032 9263047 10.2527/1997.7582024x

[B15] Herd RM Bishop SC Genetic variation in residual feed intake and its association with other production traits in British Hereford cattle Livest Prod Sci 2000 63 111 119 10.1016/S0301-6226(99)00122-0

[B16] Herd RM Arthur PF Physiological basis for residual feed intake J Anim Sci 2009 87 E64 E71 10.2527/jas.2008-1345 19028857

[B17] Wang Z Colazo MG Basarab JA Goonewardene LA Ambrose DJ Marques E Plastow G Miller SP Moore SS Impact of selection for residual feed intake on breeding soundness and reproductive performance of bulls on pastured-based multi-sire mating J Anim Sci 2012 90 2963 2969 10.2527/jas.2011-4521 22585812

[B18] Lindholm-Perry AK Kuehn LA Snelling WM Smith TPL Ferrell CL Jenkings TG Andy King D Schakelford SD Wheeler TL Freetly CH Genetic markers on BTA14 predictive for residual feed intake in beef steers and their effects on carcass and meat quality traits Anim Genet 2012 43 599 603 10.1111/j.1365-2052.2011.02307.x 22497335

[B19] Kolath WH Kerley MS Golden JW Shahid SA Johnson GS The relationships among mitochondrial uncoupling protein 2 and 3 expression, mitochondrial deoxyribonucleic acid single nucleotide polymorphisms, and residual feed intake in Angus steers J Anim Sci 2006 84 1761 1766 10.2527/jas.2005-519 16775060

[B20] Britton R Krehbiel C Nutrient metabolism by gut tissues J Dairy Sci 1993 76 2125 2131 10.3168/jds.S0022-0302(93)77547-5 8345135

[B21] Baldwin RL Modeling ruminant digestion and metabolism 1995 London: Chapman & Hall 10.1007/978-1-4899-1959-5_21 9781399

[B22] Johnson DE Johnson KA Baldwin RL Changes in liver and gastrointestinal tract energy demands in response to physiological workload in ruminants J Nutr 1990 90 0022 3166 10.1093/jn/120.6.649 2191096

[B23] Piersma T Lindström A Rapid reversible changes in organ size as a component of adaptive behaviour Trends Ecol Evol 1997 12 134 138 10.1016/S0169-5347(97)01003-3 21238009

[B24] Zitnan R Voigt J Kuhla S Wegner J Chudy A Schoenhusen U Brna M Zupcanova M Hagemeister H Morphology of small intestinal mucosa and intestinal weight change with metabolic type of cattle Vet Med-Czech 2003 53 525 532

[B25] Chappel VL Thompson MD Jeschke MG Chung DH Thompson JC Wolf SE Effects of incremental starvation on gut mucosa Digest Dis Sci 2003 48 765 769 10.1023/A:1022849112100 12741469

[B26] Dunel-Erb S Chevalier C Laurent P Bach A Decrock F Le Maho Y Restoration of the jejunal mucosa in rats refed after prolonged fasting Comp Biochem Phys A 2001 129 9330 9947 10.1016/s1095-6433(01)00360-9 11440878

[B27] Starck JM Beese K Structural flexibility of the intestine of Burmese phyton in response to feeding J Exp Biol 2001 204 325 335 11136618 10.1242/jeb.204.2.325

[B28] Baker SD Szasz JI Klein TA Kuber PS Hunt CW Glaze JB Jr Falk D Richard R Miller JC Battaglia RA Hill RA Residual feed intake of purebred Angus steers: effects on meat quality and palatability J Anim Sci 2006 84 938 945 16543572 10.2527/2006.844938x

[B29] Mader CJ Montanholi YR Wang YJ Miller SP Mandell IB McBride BW Swanson KC Relationships among measures of growth performance and efficiency with carcass traits, visceral organ mass, and pancreatic digestive enzymes in feedlot cattle J Anim Sci 2008 87 1548 1557 18952722 10.2527/jas.2008-0914

[B30] Montanholi YR Swanson KC Schenkel FS McBride BW Caldwell TR Miller SP On determination of residual feed intake and associations of infrared thermography with efficiency and ultrasound traits in beef bulls Livest Sci 2009 125 22 30 10.1016/j.livsci.2009.02.022

[B31] Getty R Sisson and Grossman’s the Anatomy of the Domestic Animals 1975 Oxford: W.B. Saunders

[B32] Carson FL Histotechnology: A self-Instructional Text 1997 Hong Kong: American Society of Clinical Pathologists

[B33] Webster AJF Ruckebusch Y, Thivend P Energy cost of digestion and metabolism in the gut Proceedings of the 5thInternational Symposium on ruminant Physiology: 3–7 September 1979; Clermont 1980 Lancaster: MTP Press 469 484

[B34] McBride BW Kelly JM Energy cost of absorption and metabolism in the ruminant gastrointestinal tract and liver: a review J Anim Sci 1990 68 2997 3010 2170320 10.2527/1990.6892997x

[B35] Cheng H Leblond CP Origin, differentiation and renewal of the four main epithelial cell types in the mouse small intestine 1. Columnar cell Am J Anat 1974 141 461 480 10.1002/aja.1001410403 4440632

[B36] Hall PA Coates PJ Ansari B Hopwood D Regulation of cell number in the mammalian gastrointestinal tract: the importance of apoptosis J Cell Sci 1994 107 3569 3577 7706406 10.1242/jcs.107.12.3569

[B37] Crosnier C Stamataki D Lewis J Organizing cell renewal in the intestine: stem cells, signals and combinatorial control Nature 2006 7 349 359 10.1038/nrg1840 16619050

[B38] Cant JP McBride BW Croom WJ Jr The regulation of intestinal metabolism and its impacts on whole animal energetics J Anim Sci 1996 74 2541 2553 8904723 10.2527/1996.74102541x

[B39] McBride BW Milligan LP The effect of lactation on ouabain-sensitive respiration of duodenal mucosa of cows Can J Anim Sci 1984 64 817 824 10.4141/cjas84-095

[B40] Burrin DG Ferrel CL Britton RA Bauer M Level of nutrition and visceral organ size and metabolic activity in sheep Brit J Nutr 1990 64 439 448 10.1079/BJN19900044 2223745

[B41] Lignot JH Helmstetter C Secor MS Postprandial morphological response of the intestinal epithelium of the Burmese python (Python molurus) Comp Biochem Phys A 2005 141 280 291 10.1016/j.cbpb.2005.05.005 16002308

[B42] Owens FN Zinn RA Kim YK Limits to starch digestion in the ruminant small intestine J Anim Sci 1986 63 1634 1648 3539905 10.2527/jas1986.6351634x

[B43] Bühler C Hammon H Rossi GL Blum JW Small intestinal morphology in eight-day-old calves fed colostrum for different durations or only milk replacer and treated with long-R3-insulin-like growth factor 1 and growth hormone J Anim Sci 1998 76 758 765 9535335 10.2527/1998.763758x

[B44] Blätter U Hammon HM Morel C Philipona C Pauprich A Romé V Le Huërou-Luron I Guilloteau P Blum JW Feeding colostrum, its composition and feeding duration variably modify enzyme activities of neonatal calves J Nutr 2001 131 1256 1263 11285335 10.1093/jn/131.4.1256

[B45] Starck JM Phenotypic plasticity, cellular dynamics, and epithelial turnover of the intestine of Japanese quail (Coturnix coturnix japonica) J Zool 1996 238 53 79 10.1111/j.1469-7998.1996.tb05379.x

[B46] Huntington GB Starch utilization by ruminants: from basics to the bunk J Anim Sci 1997 75 852 867 9078506 10.2527/1997.753852x

[B47] Burrin DG Britton RA Ferrel CL Bauer ML Level of nutrition and visceral organ protein synthetic capacity and nucleic acid content in sheep J Anim Sci 1992 70 1137 1145 1374754 10.2527/1992.7041137x

[B48] Wang YJ Holligan S Salim H Fan MZ McBride BW Swanson KC Effect of dietary crude protein level on visceral organ mass, cellularity, and the protein expression of ATP synthase, Na+/K+-ATPase, proliferating cell nuclear antigen and ubiquitin in feedlot steers Can J Anim Sci 2009 89 493 501 10.4141/CJAS08131

[B49] Wright NA Carter J Irwin M The measurements of villus cell population size in the mouse small intestine in normal and abnormal states: a comparison of absolute measurements with morphometric estimators in sectioned immersion-fixed material Cell Tissue Kinet 1989 22 425 450 2611855 10.1111/j.1365-2184.1989.tb00227.x

[B50] Iji PA Saki A Tivey DR Body and intestinal growth of broiler chicks on a commercial starter diet. 1. Intestinal weight and mucosal development Brit Poultry Sci 2001 42 505 513 10.1080/00071660120073151 11572627

[B51] McBride BW Milligan LP Influence of feed intake and starvation on the magnitude of Na,KATPase (EC 3.6.1.3) dependent respiration in duodenal mucosa of sheep Brit J Nutr 1985 53 605 614 10.1079/BJN19850070 2998448

[B52] Lobley GE Protein turnover—what does it mean for animal production? Can J Anim Sci 2001 83 327 340

[B53] Kelly JM Mutsvangwa T Milligan LP Waldo DR McBride BW Quantification of energy expenditures of gastrointestinal tract of steers fed three diets at two levels of intake Can J Anim Sci 2001 81 533 540 10.4141/A00-070

